# Differentiation of the Pea Wilt Pathogen *Fusarium oxysporum* f. sp. *pisi* from Other Isolates of *Fusarium* Species by PCR

**DOI:** 10.1264/jsme2.ME21061

**Published:** 2022-01-01

**Authors:** Shunsuke Kotera, Masashi Hishiike, Hiroki Saito, Ken Komatsu, Tsutomu Arie

**Affiliations:** 1 United Graduate School of Agricultural Science, Tokyo University of Agriculture and Technology (TUAT), 3–5–8 Saiwaicho, Fuchu, Tokyo, 183–8509, Japan; 2 Wakayama Agricultural Experiment Station, Takao, Kishigawacho, Kinokawa, Wakayama, 640–0423, Japan; 3 Institute of Agriculture, Tokyo University of Agriculture and Technology (TUAT), 3–5–8 Saiwaicho, Fuchu, Tokyo, 183–8509, Japan

**Keywords:** soilborne pathogen, detection, secreted in xylem genes (*SIX*s), pisatin demethylase gene (*PDA1*)

## Abstract

Pea wilt disease, caused by the soilborne and seedborne fungal pathogen *Fusarium oxysporum* f. sp. *pisi* (*Fop*), first appeared in Japan in 2002. We herein investigated the molecular characteristics of 16 *Fop* isolates sampled from multiple locations and at different times in Japan. The 16 isolates were divided into three clades in molecular phylogenic ana­lyses based on both the *TEF1α* gene and the rDNA-IGS region. All of the *Fop* isolates harbored a *PDA1* gene, which encodes the cytochrome P450 pisatin demethylase (Pda1), and also carried one or both of the *SIX6* and *SIX13* genes, which encode secreted in xylem (Six) proteins. Other forms of *F. oxysporum* and other species of *Fusarium* did not carry these sets of genes. Based on these results, a PCR method was developed to identify *Fop* and differentiate it from other forms and non-pathogenic isolates of *Fusarium* spp. We also demonstrated that the PCR method effectively detected *Fop* in infected pea plants and infested soils.

Pea (*Pisum sativum* L.) is one of the most commonly and widely cultivated Fabaceae plants. In 2019, 21.8 million tons of green peas and 14.2 million tons of dry peas were harvested worldwide (FAOSTAT, 2019). In Japan,‍ ‍20,000 tons of podded green peas, 6,300 tons of green peas, and 900 tons of dry peas were harvested in 2019 (e-Stat, 2019). Similar to many other crops and vegetables, diseases threaten pea cultivation by decreasing production. Harveson *et al.* (2020) listed 27 pea diseases caused by fungi, bacteria, viruses, and nematodes, and The Phytopathological Society of Japan (2021) listed 30 pea diseases.

*Fusarium oxysporum* Schlecht. emend. Snyd. et Hans. f. sp. *pisi* (Lindf.) Snyd. et Hans. (*Fop*) causes Fusarium wilt, one of the most destructive diseases of pea (Kraft, 1994; Haglund and Kraft, 2001). Pea plants infected by this pathogen present with leaf yellowing, browning of the vascular tissues, and blight and ultimately die (Matsusaki *et al.*, 2003). Pea wilt was initially reported in the USA in 1925, and also occurs in Europe, and Asia (Kraft, 1994). However, it was not detected in Japan until 2002 (Sakoda *et al.*, 2018, 2019). The Plant Protection Station of the Japanese Ministry of Agriculture, Forestry and Fisheries (MAFF) has listed *Fop* as one of the pathogens that needs to be monitored to prevent invasion (MAFF, 2021). Since its first appearance in Aichi Prefecture in 2002, sporadic outbreaks of *Fop* have been reported in several areas of Japan, including Shizuoka, Hokkaido, and Wakayama Prefectures (Sakoda *et al.*, 2018). Countermeasures for eradication have been taken in each area. It is extremely important to develop methods for the specific identification of *Fop* so that it may be identified and eradicated from infested fields.

*F. oxysporum* is a ubiquitous ascomycete fungus that is widely distributed in the environment, and many strains are known to be soilborne and/or seedborne pathogens of plants. The range of plant species that may be infected (the host range) of each isolate is strictly and clearly defined for this fungus, and more than 120 forms (formae speciales; ff. spp.) have been identified based on their host ranges (Michielse and Rep, 2009; Kashiwa *et al.*, 2016; Arie, 2019). One of the forms that causes pea wilt is f. sp. *pisi* (*Fop*), which never causes disease in other plant species. The detection of *Fop* in soil or plant tissues and its differentiation from other forms and non-pathogenic isolates of *F. oxysporum* and *Fusarium* spp. are important for its eradication.

Although the specific identification of *Fop* is possible using *in planta* bioassays based on the inoculation of pea plants, this process requires too much time, space, and labor. Therefore, faster, easier, and more accurate techniques to identify *Fop* are needed. Recent molecular and genomic studies have begun to reveal the mechanisms underlying host specificity in *F. oxysporum* as well as the factors influencing host specificity, such as secreted proteins called effectors, which may be employed to discriminate between pathogenic forms (Arie, 2019, 2020). For example, PCR, real-time PCR, and Loop-mediated isothermal amplification (LAMP) methods that target effector genes have been developed for the specific identification of *F. oxysporum* f. sp. *lycopersici*, which is the form that causes tomato wilt (Lievens *et al.*, 2009; Inami *et al.*, 2010; Ayukawa *et al.*, 2016, 2017; Kashiwa *et al.*, 2016).

Effectors are proteins secreted by plant pathogens during host colonization and are essential for pathogenicity. The presence/absence patterns of effector genes may sometimes influence host specificity in *F. oxysporum* (van Dam *et al.*, 2016). Lineage-specific (LS) chromosomes, which are not necessary for fungal growth, are rich in genes encoding effectors. LS chromosomes have been identified in *F. oxysporum* ff. spp. *lycopersici* and *radicis-cucumerinum*, the cucumber root and stem rot pathogen (Ma *et al.*, 2010; van der Does *et al.*, 2016; Ayhan *et al.*, 2018). Some of the Secreted in xylem (Six) proteins (Six1 to Six14) have been identified as effectors, and their encoding genes, *SIX1* to *SIX14*, are located on LS chromosomes in *F. oxysporum* f. sp. *lycopersici* (Schmidt *et al.*, 2013; Vlaardingerbroek *et al.*, 2016). Moreover, homologs of the *SIX* genes have been identified in various pathogenic forms of *F. oxysporum*, including *Fop* (Houterman *et al.*, 2009; Gawehns *et al.*, 2014; Taylor *et al.*, 2016; Jenkins *et al.*, 2021).

Plants produce antibiotic chemicals such as phytoanticipins and phytoalexins, which are involved in innate and acquired resistance against pathogens (VanEtten *et al.*, 2001). Some pathogenic forms of *Fusarium* spp. harbor enzymes that detoxify phytoanticipins or phytoalexins (VanEtten *et al.*, 1994; Curir *et al.*, 2005; Coleman *et al.*, 2011; Milani *et al.*, 2012). The pea root rot pathogen *F. solani* f. sp. *pisi* (*Fsp*) possesses the *PDA1* gene, which encodes the cytochrome P450 pisatin demethylase (Pda1) that detoxifies pisatin, a phytoalexin produced by pea. Pda1 is a crucial factor that influences both the pathogenicity and host specificity of *Fsp* (VanEtten *et al.*, 1998; Miao *et al.*, 1991; Bani *et al.*, 2014). Coleman *et al.* (2011) reported that *Fop* also carries a *PDA1* gene. The isolate NRRL 26761 of *F. oxysporum* f. sp. *phaseoli*, the yellow pathogen of common bean (*Phaseolus vulgaris* L.), harbors a *PDA1* homolog and is pathogenic to pea (Coleman *et al.*, 2011). These findings suggest the importance of *PDA1* for the pathogenicity of *Fop* in pea.

In the present study, we performed a phylogenetic ana­lysis of Japanese *Fop* isolates using two genetic regions: the *translation elongation factor 1α* gene (*TEF1α*) and the ribosomal DNA intergenic spacer (rDNA-IGS) region. We used PCR to investigate the presence/absence of the *SIX*s and *PDA1* genes, and developed a PCR-based method to identify *Fop* and distinguish it from other forms of *F. oxysporum* and other *Fusarium* isolates.

## Materials and Methods

### Fungal isolates

The *Fusarium* isolates used in the present study are listed in Table 1. The total number of Japanese *F. oxysporum* f. sp. *pisi* (*Fop*) isolates examined herein was 16. Among these isolates, 15 were obtained from the Yokohama Plant Protection Station (YPPS), MAFF, Yokohama, Japan, which included five isolates from Aichi Prefecture, three from Shizuoka, one from Hokkaido, and six from Wakayama. The isolate (200929a) examined in the present study was isolated from a pea plant with wilt symptoms in a Wakayama field. All *Fop* isolates were obtained through single colony selections. K3-1, K3-3, K4-1, K4-2, and K5-2 were isolated from pea seeds at the YPPS. Since they did not exhibit pathogenicity in peas, they were defined as non-pathogenic isolates (Table 1). Isolates were cultured and maintained on potato dextrose agar (PDA) medium plates at 28°C under dark conditions. Isolates were also stored in 25% (v/v) glycerol at –80°C.

### *In planta* pathogenicity assays using pea plants

Regarding *in planta* pathogenicity assays, we used 26 isolates of *Fusarium* spp. including the 16 *Fop* isolates (Table 1). Each isolate was cultured in potato dextrose broth (PDB) medium for 5 days at 28°C with reciprocal shaking at 120‍ ‍rpm. The bud cells that formed were filtered through a double layer of sterilized cheese cloth to remove mycelia, collected by centrifugation at 3,000×*g* for 10‍ ‍min, and suspended in sterilized water at a concentration of 1.0×10^7^‍ ‍cells‍ ‍mL^–1^. This suspension was used as the inoculum.

To test the pathogenicity of each isolate, we employed the soil drenching method with the pea cultivar Misasa (Asahi Noen Seed), which is susceptible to *Fop* (Sakoda *et al.*, 2018). Two seeds were sown in each plastic pot with a diameter of 7‍ ‍cm containing autoclaved (121°C, 40‍ ‍min) soil (Kumiai Nippi Engeibaido No. 1; Nihon Hiryo). The roots of each 10-day-old pea plant were wounded by inserting a plastic peg into the soil five times, and the inoculum was then added to the soil at a rate of 1‍ ‍mL plant^–1^. After inoculation, plants were maintained in a greenhouse at 28°C. Tests were conducted using four or six plants with three biological replications. Disease severity in each plant at 28 days post-inoculation was evaluated as follows: 0, no symptoms; 1, yellowing or wilting of the lower leaves; 2, yellowing or wilting of the upper and lower leaves; 3, wilting of the entire plant; 4, death.

### Extraction of fungal genomic DNA (gDNA)

gDNA was extracted from mycelia that had been cultured on a PDA plate using the procedure of Saitoh *et al.* (2006). A Nanodrop One Spectrophotometer (Thermo Fisher Scientific) was employed to assess the concentration and quality of gDNA.

### Identification of mating types by PCR

The mating type of each isolate was identified by PCR using a MiniAmp Thermal Cycler (Thermo Fisher Scientific) with the primers listed in Table S1. The method and PCR conditions employed were identical to those described by Inami *et al.* (2012). Isolates from which an approximately 280-bp fragment was amplified with the primer set Gfmat1a/Gfmat1b were identified as MAT1-1. Isolates from which an approximately 220-bp fragment was amplified with the primer set GfHMG1/GfHMG2 were identified as MAT1-2.

### PCR amplification of the *TEF1α* gene fragment and the rDNA-IGS region

In the molecular phylogenetic ana­lysis, we amplified fragments of the *TEF1α* gene (*ca.* 700 bp) and the rDNA-IGS region (*ca.* 600 bp) from each isolate using the EF1/EF2 primers for *TEF1α* (O’Donnell *et al.*, 2009) and the FIGS11/FIGS12 primers for the rDNA-IGS region (Kawabe *et al.*, 2005) (Table S1). We used a MiniAmp Thermal Cycler (Thermo Fisher Scientific), and 10‍ ‍μL of the PCR mixture contained 30‍ ‍ng gDNA, 1×Ex *Taq* Buffer (Takara Bio), 0.5‍ ‍mM of each dNTP (Takara Bio), 0.2‍ ‍μM of each primer, and 0.5‍ ‍U TaKaRa Ex *Taq* (Takara Bio). Reactions consisted of three steps: 94°C for 1‍ ‍min; 30 cycles of 94°C for 30‍ ‍s, 58°C for 30‍ ‍s, and 72°C for 1‍ ‍min; and 72°C for 7‍ ‍min.

### Sanger sequencing

PCR amplicons were sequenced directly and after cloning. Regarding direct sequencing, each amplicon was purified with ExoSAP-IT (Thermo Fisher Scientific) and sequenced in a 3710xl Genetic Analyzer (Thermo Fisher Scientific) using the BigDye Terminator v3.1 Cycle Sequencing Kit (Thermo Fisher Scientific) with the primers used for amplification. Concerning cloning, each amplicon was ligated into the pGEM-T Easy vector (Promega). The inserted DNA fragments were then sequenced with the M13F/M13R primers (5′-CGCCAGGGTTTTCCCAGTCACGAC-3′/5′-AGCGGATAACAATTTCACACAGGA-3′). Data were processed using GENETYX-Mac version 11.2.1 software (Genetyx) and deposited in GenBank (Table 1 and 2).

### Phylogenic ana­lyses

The phylogenic relationships between the Japanese and non-Japanese *Fop* isolates were analyzed using *TEF1α* sequences (Table 1 and S2). To clarify the phylogenic positions of the Japanese *Fop* isolates among various other forms of *F. oxysporum*, we performed a phylogenetic ana­lysis using rDNA-IGS sequences (Table 1). Multiple sequences were aligned using ClastalW version 2.1 (Larkin *et al.*, 2007), and phylogenetic ana­lyses were performed using the maximum likelihood method. We adopted the Hasegawa-Kishino-Yano model (Hasegawa *et al.*, 1985) with 1,000 replicates of bootstrap values. The outgroup for *TEF1α* was the root rot pathogen of pea, *F. solani* f. sp. *pisi* isolate C1-2A (Table 1), while that for rDNA-IGS was *F. sacchari* isolate FGSC 7610 (Table 1). All evolutionary ana­lyses were performed using MEGA X software (Kumar *et al.*, 2018; Stecher *et al.*, 2020).

### Detection of *SIX* and *PDA1* genes by PCR

All 16 Japanese *Fop* isolates were subjected to PCR ana­lyses aimed at detecting homologs of the *F. oxysporum* f. sp. *lycopersici SIX* genes and *PDA1* using previously designed primer sets (Table S1; van der Does *et al.*, 2008; Lievens *et al.*, 2009; Meldrum *et al.*, 2012; Milani *et al.*, 2012; Taylor *et al.*, 2016). Ten microliters of the PCR mixture contained 30‍ ‍ng gDNA, 1×Ex *Taq* Buffer, 0.5‍ ‍mM of each dNTP, 0.2‍ ‍μM of each primer, and 0.5‍ ‍U Ex *Taq*. The reaction conditions for *SIX1* to *SIX5*, *SIX7*, and *SIX9* to *SIX14* were as follows: 94°C for 1‍ ‍min; 30 cycles of 94°C for 30‍ ‍s, 58°C for 30‍ ‍s, and 72°C for 1‍ ‍min; and 72°C for 7‍ ‍min. The conditions for *SIX6* were as follows: 94°C for 1‍ ‍min; 30 cycles of 94°C for 30‍ ‍s, 50°C for 30‍ ‍s, and 72°C for 45 s; and 72°C for 7‍ ‍min. The conditions for *SIX8* were as follows: 94°C for 1‍ ‍min; 30 cycles of 94°C for 30‍ ‍s, 58°C for 30‍ ‍s, and 72°C for 30 s; and 72°C for 7‍ ‍min. The conditions for *PDA1* were as follows: 94°C for 1‍ ‍min; 30 cycles of 94°C for 30‍ ‍s, 63°C for 30‍ ‍s, and 72°C for 1‍ ‍min; and 72°C for 7‍ ‍min.

In reactions designed to detect *Fop* isolates using the primers listed in Table 3, 10‍ ‍μL of the PCR mixture contained 30‍ ‍ng of fungal gDNA, 1×PCR Buffer for KOD Fx Neo (Toyobo), 0.4‍ ‍mM of each dNTP, 0.1‍ ‍μM of each primer, and 0.2‍ ‍U KOD Fx Neo (Toyobo). PCR conditions were 94°C for 1‍ ‍min, 30 cycles of 98°C for 10‍ ‍s, 60°C for 30‍ ‍s, and 68°C for 30‍ ‍s, followed by 68°C for 7‍ ‍min.

### Detection limits of piPDA, piSIX6, and piSIX13 primer sets with *Fop* gDNA

To clarify the detection limits of the primers listed in Table 3, the gDNA of *Fop* isolate 39b was serially diluted with water, with concentrations ranging between 3‍ ‍ng μL^–1^ and 30 fg μL^–1^. Diluted samples (1‍ ‍μL per reaction) were used as templates in PCR reactions set up as described above.

### Detection of *Fop* in infected plants and infested soils

Pea plants (cv. Misasa) were inoculated with *Fop* isolate 39b as described above for the pathogenicity tests. A sterilized toothpick was inserted into the basal stem tissues 28 days after the inoculation and then soaked in the PCR mixture for 5 s. Two samples from two individual plants each were used. Healthy pea plants were employed as the control.

To prepare an artificially infested soil with *Fop* isolate 39b, 5‍ ‍g of autoclaved soil was mixed with 1‍ ‍mL of the bud cell suspension (1.0×10^7^‍ ‍cells‍ ‍mL^–1^) in a Petri dish (90‍ ‍mm in diameter). The infested soil was dried at room temperature overnight. A similar sample was prepared with distilled water as a negative control. Soil DNA was extracted from 0.5‍ ‍g of each soil sample using the FastDNA SPIN Kit for Soil (MP Biomedicals) with 10% skim milk (w/v) in a Fastprep-24 grinder (MP Biomedicals), as previously described by Kashiwa *et al.* (2016). Fifty nanograms of soil DNA was used as the template for PCR. Two replicates were prepared for each treatment.

Soil samples (original) were collected from two different pea-growing fields (No. 28 and 49) in May 2020. Both fields had histories of pea wilt disease; however, the occurrence of pea wilt was not confirmed in the 2019 crop season (between September 2019 and May 2020). Fields No. 28 and 49 were both subsequently disinfested using soil solarization and chloropicrin-fumigation, and soil samples were again collected (disinfested; September 2020). Five grams of soil sampled from three locations in each field were mixed well and 15‍ ‍g of soil was subjected to soil DNA extraction as described above.

## Results

### Pathogenicity in peas

All 16 *Fop* isolates showed pathogenicity in pea cv. Misasa (Table 1 and Fig. S1). As expected, the isolate C1-2A of *F. solani* f. sp. *pisi* (*Fsp*) exhibited strong pathogenicity in peas (Table 1 and Fig. S1). It was not possible to distinguish between the symptoms presented by *Fop* and *Fsp*. We also tested *F. oxysporum* f. sp. *adzukicola* (the pathogen of adzuki bean wilt) isolate 241054, *F. oxysporum* f. sp. *spinaciae* (the pathogen of spinach wilt) isolate 170612b, non-pathogenic *F. oxysporum* isolates K3-1, K3-2, K4-1, K4-2, and K5-2 from pea, *F. solani* isolate 305125 from sweet pea (*Lathyrus odoratus* L.), and *F. commune* isolate W5 from rice (*Oryza sativa* L.). None of these isolates exhibited any pathogenicity in peas (Table 1 and Fig. S1).

### Mating type

We identified the mating type of each isolate and found that 15 out of the 16 Japanese *Fop* isolates were MAT1-2 (Table 1). Only one isolate, 9-1-M-2 from Shizuoka Prefecture, was MAT1-1 (Table 1 and Fig. 2). All isolates were MAT1-1 or MAT1-2, which suggested that all of the tested isolates were heterothallic (Table 1; Arie *et al.*, 2000).

### Phylogeny

**Fig. 1.**
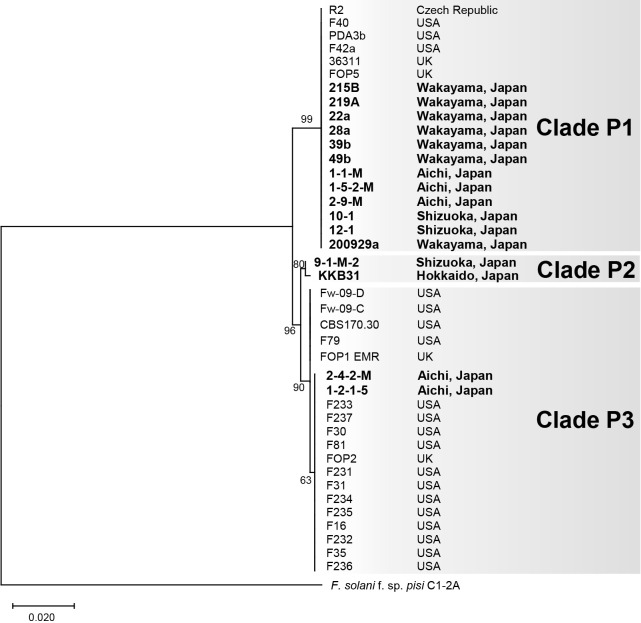
Phylogenetic tree of *Fusarium oxysporum* f. sp. *pisi* based on the *TEF1α* gene. The tree was constructed using the maximum likelihood method and the Hasegawa-Kishino-Yano model. Numbers on nodes represent bootstrap values estimated from 1,000 replicates, where bootstrap values are higher than 60%. *Fop* isolates were divided into three clades, P1, P2, and P3. *F. solani* f. sp. *pisi* isolate C1-2A was used as an outgroup. The source location of each isolate is shown on the right of the phylogenetic tree. Sequence information is presented in Table 1 and S2.

We constructed a phylogenetic tree based on *TEF1α* sequences from the 16 Japanese *Fop* isolates and 24 *Fop* isolates from other countries whose sequences were available in the NCBI database (Table S2). The tree supported three clades, P1–P3 (Fig. 1). Clade P1 comprised 18 isolates, including all seven isolates from Wakayama, three from Aichi, and two from Shizuoka, along with three isolates from the USA, two from the UK, and one from the Czech Republic. Clade P2 was composed of two isolates, one from Shizuoka and one from Hokkaido. Clade P3 included two Japanese isolates, 16 isolated from the USA, and two from the UK.

**Fig. 2.**
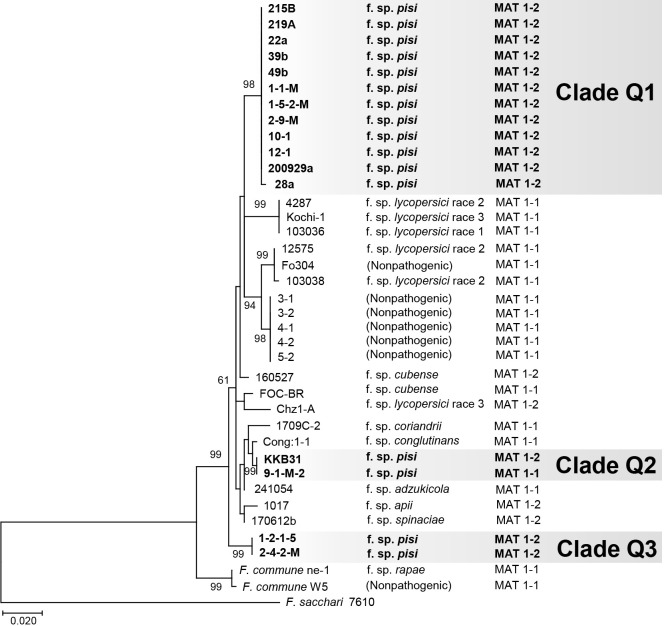
Phylogenetic tree of *Fusarium oxysporum* isolates based on the rDNA-IGS region. The tree was constructed using the maximum likelihood method and the Hasegawa-Kishino-Yano model. Numbers on nodes represent bootstrap values estimated from 1,000 replicates when bootstrap values are higher than 60%. *Fop* isolates were divided into three clades, Q1, Q2, and Q3. *F. sacchari* isolate FGSC 7610 was used as an outgroup. The form and mating type of each *Fusarium* isolate is shown on the right of the tree. Sequence information is presented in Table 1.

We also constructed a phylogenic tree based on the rDNA-IGS sequences from the 16 Japanese *Fop* isolates, 14 isolates of other forms of *F. oxysporum* and *F. commune*, and seven non-pathogenic *F. oxysporum* and *F. commune* isolates, five of which were isolated from pea (Fig. 2). In this tree, the 16 Japanese *Fop* isolates again formed three well supported clades, Q1–Q3, corresponding to clades P1–P3, respectively, in the *TEF1α* phylogeny (Fig. 1). Clades P1 and Q1 contained 12 Japanese *Fop* isolates that were all MAT1-2, clades P2 and Q2 contained the MAT1-2 isolate KKB31 and the MAT1-1 isolate 9-1-M-2, and clades P3 and Q3 contained the MAT1-2 isolates 1-2-1-5 and 2-4-2-M (Fig. 1, 2, and Table 1).

### Presence or absence of *SIX* and *PDA1* genes

We used PCR with primers designed to amplify the 14 *SIX* genes of *F. oxysporum* f. sp. *lycopersici* in order to investigate the presence or absence of *SIX* homologs in the 16 Japanese *Fop* isolates, the other forms of *F. oxysporum*, the non-pathogenic *F. oxysporum* isolates, and the other *Fusarium* spp. isolates listed in Table 2. In this ana­lysis, we used the previously designed primers listed in Table S1. Among the 16 *Fop* isolates, 12 possessed *SIX6* and *SIX13* homologs (Table 2). Isolates 1-2-1-5 and 2-4-2-M had homologs of *SIX14* as well as *SIX*s *6* and *13*. Isolate 9-1-M-2 had homologs of *SIX*s *7*, *8*, and *10–14*, but lacked *SIX6*. KKB31 possessed *SIX6*, but lacked *SIX13*. *F. oxysporum* f. sp. *cubense* isolates also had both *SIX6* and *SIX13*, and the *F. oxysporum* f. sp. *azdukicola* isolate possessed *SIX13*. As expected, the *F. oxysporum* f. sp. *lycopersici* isolates had most or all of the *SIX* genes. The other forms and non-pathogenic isolates did not carry homologs that were detectable by the primers used (Table 2). Moreover, we did not detect *SIX* homologs in *Fsp*, the root rot pathogen of pea (Table 2). We also employed PCR and previously designed *PDA1* primers (Table S1) to search for *PDA1* homologs in all the isolates listed in Table 2. In this case, *PDA1* homologs were only detected in the 16 *Fop* isolates, *Fsp*, and an isolate (170612b) of *F. oxysporum* f. sp. *spinaciae* (Table 2). Therefore, we found that the *Fop* isolates harbored *SIX6* and/or *SIX13* together with *PDA1*.

### Differentiation of *Fop* isolates from other *F. oxysporum* isolates and *Fusarium* spp. by PCR

As demonstrated in the phylogenetic study, *Fop* is polyphyletic among *F. oxysporum* isolates (Fig. 2). Therefore, the primer sets for the rDNA-IGS sequence may not be applicable for the differentiation of *Fop* from other forms and non-pathogenic isolates of *F. oxysporum*. This is consistent with previous findings on other forms, including *F. oxysporum* ff. spp. *lycopersici*, *cubense*, and *apii* (the celery wilt pathogen) (O’Donnell *et al.*, 1998; Kawabe *et al.*, 2005; Epstein *et al.*, 2017).

On the other hand, all *Fop* isolates tested carried either or both of the *SIX6* and *SIX13* homologs together with the *PDA1* gene, and among the isolates tested in Table 2, *Fop* isolates were the only ones to carry this combination of three genes (*PDA1*; *SIX6* and/or *SIX13*). Therefore, we designed specific primer sets (piPDA, piSIX6, and piSIX13) targeting these genes (Table 3). The primer sets were designed to have the same annealing temperature (60°C) in order to obtain the desired amplificons under the same reaction conditions. Regarding the specific detection of *Fop* by PCR, we employed KOD Fx Neo polymerase, which may be used with crude DNA samples.

**Fig. 3.**
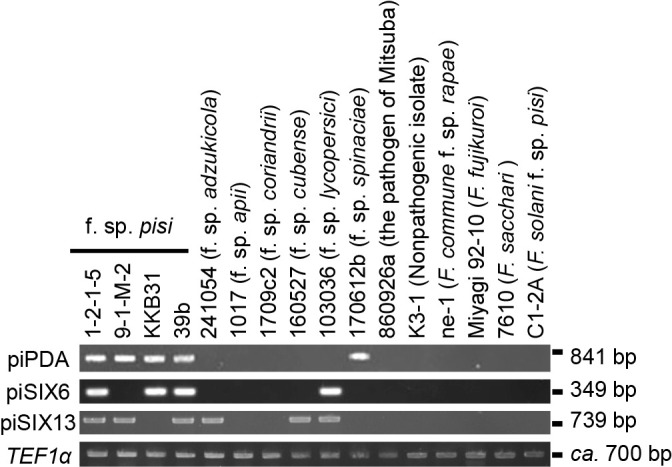
Differentiation of *Fusarium oxysporum* f. sp. *pisi* from other isolates of *Fusarium* species by PCR. PCR was performed using representative isolates and the primer sets piPDA, piSIX6, piSIX13, and *TEF1α* (Table 3 and S1). Products of 841, 349, 739, and approximately 700 bp were generated with primer sets piPDA, piSIX6, piSIX13, and *TEF1α*, respectively.

The piPDA primer set amplified a fragment (841 bp) of *PDA1* from all of the *Fop* isolates and from an isolate of* F. oxysporum* f. sp. *spinaciae* (Fig. 3 and Table 4). However, no amplicons were obtained from any of the other *Fusarium* isolates, including C1-2A of *Fsp*, which carries a *PDA1* gene that is not targeted by the specific sequences of the piPDA primers (Fig. 3 and Table 4). The piSIX6 primer set amplified a fragment (349 bp) from all the *F. oxysporum* f. sp. *lycopersici* isolates, and all the *Fop* isolates, except for 9-1-M-2, but did not amplify the fragment from any of the other *Fusarium* isolates (Fig. 3 and Table 4). Similarly, the piSIX13 primer set amplified a fragment (739 bp) from an isolate of *F. oxysporum* f. sp. *adzukicola*, both of the isolates of *F. oxysporum* f. sp. *cubense*, all of the *F. oxysporum* f. sp. *lycopersici* isolates, and all of the *Fop* isolates, except for KKB31, but did not amplify the fragment from any other *Fusarium* isolates (Fig. 3 and Table 4). Therefore, as shown in Table 4, only *Fop* isolates showed positive results with the piPDA primers plus one or both of the piSIX6 and piSIX13 primers.

### Detection limits of piPDA, piSIX6, and piSIX13 primers

**Fig. 4.**
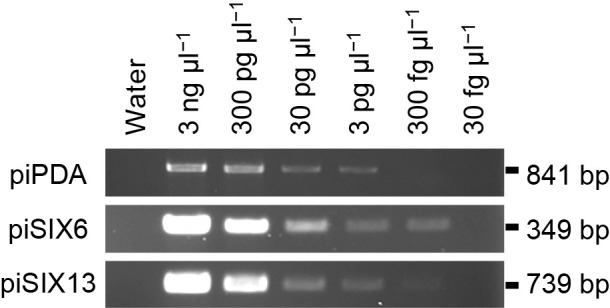
Assessment of detection limits of piPDA, piSIX6, and piSIX13 primer sets. gDNA from *Fop* isolate 39b was serially diluted from 3‍ ‍ng μL^–1^ to 30 fg μL^–1^, and each dilution was used as the template in PCRs with the piPDA, piSIX6, and piSIX13 primer sets.

To clarify the detection limits of the primer pairs designed in the present study, we made serial dilutions of gDNA from isolate 39b and used them in PCRs with the three primer sets. The piPDA primer set detected as low as 3 pg μL^–1^ of gDNA, while the piSIX6 and piSIX13 primer sets detected as low as 300 fg μL^–1^ (Fig. 4).

### Detection of *Fop* in infected pea plants

**Fig. 5.**
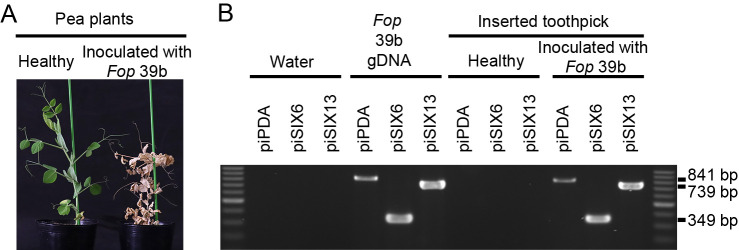
Detection of *Fusarium oxysporum* f. sp. *pisi* in infected plant tissues by direct PCR. (A) Photographs of the pea plants used. Healthy pea plants were inoculated with sterilized water and infected pea plants were inoculated with *Fop* isolate 39b. (B) PCR was performed using sterile water, the gDNA of *Fop* isolate 39b, and material from each plant as templates. The primer sets piPDA, piSIX6, and piSIX13 were used. To pick a small amount of material from each plant, a toothpick was inserted into basal stem tissues and soaked in the PCR mixture as a template.

To investigate whether the piPDA, piSIX6, and piSIX13 primer sets are applicable for the detection of *Fop* in infected plants, we inoculated pea (cv. Misasa) with *Fop* isolate 39b, and a small amount of the infected plant was picked 28 days later by inserting a sterilized toothpick into the basal stem tissues. The toothpick was then soaked in PCR mixture to provide template DNA for the reaction. Healthy pea plants were used as controls. The pea plants inoculated with *Fop* produced positive bands of the expected sizes in PCRs with the three primer sets. On the other hand, no bands were produced in PCRs with the healthy pea plants. Representative data are shown in Fig. 5.

### Detection of *Fop* in soil samples

**Fig. 6.**
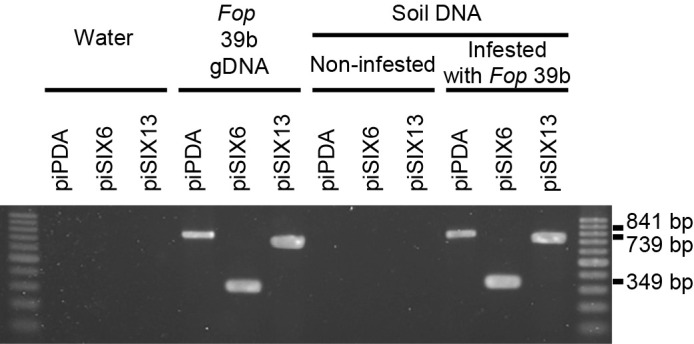
Detection of *Fusarium oxysporum* f. sp. *pisi* in artificially infested soil by PCR. PCRs were performed using sterile water, the gDNA of *Fop* isolate 39b, and DNA extracted from non-infested soil and soil infested with 39b as templates. The primer sets piPDA, piSIX6, and piSIX13 were used.

We performed PCRs using the DNAs from artificially infested soil with the three primer sets piPDA, piSIX6, and piSIX13. The DNAs extracted from *Fop*-infested soil produced positive bands of the expected sizes with all three primer sets (Fig. 6). On the other hand, no bands were produced in PCRs with the DNAs extracted from non-infested soil. Representative data are shown in Fig. 6.

**Fig. 7.**
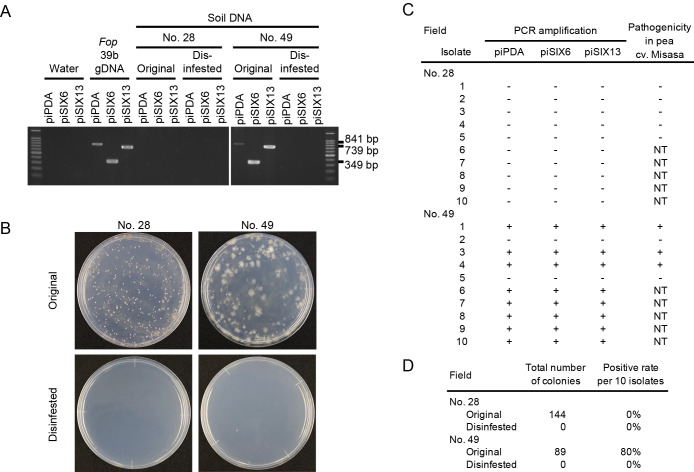
Detection of *Fusarium oxysporum* f. sp. *pisi* in soil from pea fields by PCR. (A) PCR was performed using soil DNA extracted from pea fields No. 28 and No. 49, before (original) and after sterilization (disinfested), as templates. As negative and positive controls, sterile water and the gDNA of *Fop* isolate 39b, respectively, were applied as templates. The primer sets piPDA, piSIX6, and piSIX13 were used. (B) Colonies on *F. oxysporum*-selective medium plates. Photographs were taken 5 days after inoculation with diluted soil samples collected from fields No. 28 and No. 49, before (original) and after sterilization (disinfested). (C) PCR was performed using the primer sets piPDA, piSIX6, and piSIX13 with gDNA extracted from 10 randomly selected *F. oxysporum* isolates from fields No. 28 and No. 49. Positive and negative amplifications are shown by + and –, respectively, in PCR. Pathogenicity, no pathogenicity and not tested (NT) are shown by +, –, and NT, respectively, for pathogenicity in pea cv. Misasa. (D) The total numbers of colonies isolated from the diluted soil samples from fields No. 28 and No. 49, and the percentages of PCR-positive results from the 10 randomly selected isolates from each pea field.

DNAs from the soil samples of two pea-growing fields (No. 28 and 49) were extracted. PCRs using soil DNAs with the piPDA, piSIX6, and piSIX13 primers amplified bands of the expected sizes from the original field No. 49 sample, but not from the No. 28 sample (Fig. 7A). This result suggested that *Fop* existed in the field No. 49 sample.

We then applied 10-fold dilutions (w/w) of each soil mixture to plates containing the *F. oxysporum*-selective medium Fo-G1 (Nishimura, 2007). In total, 144 and 89 isolates were obtained from fields No. 28 and 49, respectively (Fig. 7B and D). gDNAs were extracted from the 10 isolates randomly selected as representative isolates of each field, and subjected to PCRs with the piPDA, piSIX6, and piSIX13 primers. All 10 isolates from field No. 28 were negative. On the other hand, 8 out of 10 isolates from field No. 49 were positive for the three primer sets (Fig. 7C and D). PCRs were also performed on disinfested No. 28 and 49 soil samples. No bands were obtained from the disinfested samples of either field (Fig. 7A). Moreover, no colonies were detected from disinfested soils with *F. oxysporum*-selective medium (Fig. 7B and D).

## Discussion

Phylogenetic relationships among Japanese and other *Fop* isolates were examined in the present study, and the presence or absence of the putative effector genes *SIX1–14* and *PDA1* in Japanese *Fop* isolates was also investigated. Moreover, a PCR-based technique for identifying *Fop* and differentiating it from other *F. oxysporum* forms and other *Fusarium* spp. was established. The PCR method effectively detected *Fop* in infected pea plants and infested soils.

Both phylogenetic trees based on *TEF1α* and the rDNA-IGS region showed that *Fop* isolates fell into three independent clades (P1–3 in Fig. 1 and Q1–3 in Fig. 2). Clades P1, P2, and P3 each contained the same isolates as clades Q1, Q2, and Q3, respectively. These phylogenetic relationships suggest the polyphyletic origin of *Fop*. This is consistent with previous findings reported by Kawabe *et al.* (2005) and Epstein *et al.* (2017), showing that *F. oxysporum* ff. spp. *lycopersici* and *apii*, respectively, were polyphyletic and difficult to distinguish from other forms based on phylogenies. On the other hand, the phylogenetic tree based on *TEF1α* (Fig. 1), which shows relationships among Japanese and non-Japanese *Fop* isolates, indicated that the isolates in clades P1 and P3 were closely related to those from the USA, UK, and Czech Republic. All of the Wakayama isolates were in clade P1 together with the isolates from other countries, suggesting that the Wakayama isolates are monophyletic and arrived from other countries via seeds.

*F. oxysporum* has been suggested to carry functional mating type genes despite being an asexual fungus (Arie *et al.*, 2000). Kawabe *et al.* (2005) reported that the *F. oxysporum* f. sp. *lycopersici* isolates belonging to each phylogenetic group carry identical mating type genes, and suggested that asexual reproduction is a major driving force for diversification. All of the *Fop* isolates belonging to clades Q1 and Q3 are MAT1-2, while the two isolates 9-1-M-2 and KKB31 in clade Q2 have different mating types (MAT1-1 and MAT1-2, respectively), suggesting that these two isolates reproduce sexually (Fig. 2). However, our attempts to cross these isolates (9-1-M-2 as MAT1-1 and KKB31 as MAT1-2) on carrot medium using the method described by Leslie and Summerell (2006) have so far been unsuccessful (data not shown).

All twelve Japanese isolates belonging to clade P1 carry both the *SIX6* and *SIX13* genes (Table 2). The two isolates in clade P3 carry *SIX6*, *SIX13*, and *SIX14* (Table 2). The two isolates in clade P2 carry different combinations of *SIX*s as follows: KKB31 carries *SIX6*, and 9-1-M-2 carries *SIX*s *7*, *8*, and *10–14* (Table 2). Taken together, these results show that *Fop* isolates carry both *SIX6* and/or *SIX13*. Six6 is a cysteine-rich protein with a signal peptide that functions to suppress *I2* resistance in tomato (Gawehns *et al.*, 2014). *SIX6* was initially reported in *F. oxysporum* f. sp. *lycopersici* (Lievens *et al.*, 2009) and was suggested to be involved in, but not essential for pathogenicity (Gawehns *et al.*, 2014; Vlaardingerbroek *et al.*, 2016). On the other hand, *SIX6* disruptants in *F. oxysporum* f. sp. *radicis-cucumerinum* reduced their pathogenicity to cucumber, suggested that *SIX6* played an important role in pathogenicity (van Dam *et al.*, 2017). Six13 is also a cysteine-rich protein with a signal peptide; however, its function in pathogenicity in *F. oxysporum* currently remains unclear. Further studies are warranted to clarify whether *SIX6* and *SIX13* are involved in pathogenicity to pea in *Fop* and complement each other.

*PDA1* encodes a pisatin demethylase that degrades pisatin, a fungicidal chemical produced by pea plants. Therefore, it is reasonable that all of the *Fop* isolates tested in the present study carry *PDA1*. This is consistent with previous findings showing that when *PDA1* was introduced into *F. oxysporum* f. sp. *lini*, the flax wilt pathogen, it acquired pathogenicity to pea (Coleman *et al.*, 2011). *Fsp*, the pathogen of the root rot of pea, also has a *PDA1* gene (Table 2). *F. oxysporum* f. sp. *spinaciae* (170612b), the wilt pathogen of spinach, also carries a *PDA1* gene with high sequence homology (Table 2); however, this isolate is not pathogenic to pea (Fig. S1). Spinach (*Spinacia oleracea* L.) does not produce pisatin. Therefore, the Pda1 of *F. oxysporum* f. sp. *spinaciae* may play another role other than the demethylation of pisatin.

We found that *Fop* isolates differentiated from other forms and non-pathogenic *F. oxysporum* isolates because they uniquely carry *PDA1* along with *SIX6* and/or *SIX13*. Therefore, we designed the primer sets piPDA, piSIX6, and piSIX13 (Table 3), and successfully established a PCR method to specifically identify *Fop*. Based on our study of the detection limits of PCR, the threshold of detection was at least 3 pg μL^–1^ of gDNA (Fig. 4). We demonstrated that the PCR method may be used to detect *Fop* in infected pea plant tissues and soils infested with *Fop*.

We successfully detected *Fop* within 3 h by simply transferring a small amount of infected plant tissues into the PCR mixture using a toothpick (Fig. 5). Therefore, it was possible to eliminate the steps of isolation, cultivation, and gDNA extraction from the fungus for the detection of *Fop*. This method will allow for the rapid diagnosis of pea wilt in the field.

We successfully detected *Fop* by PCR using soil DNAs from pea-growing fields as templates (Fig. 7). It is important to note that we detected *Fop* even from a field (No. 49) in which pea wilt disease was not observed in the previous crop (Fig. 7A and C). This result suggests that *Fop* was present in the soil at a density lower than that needed to cause disease in pea plants. After the soil solarization and chloropicrin fumigation of fields No. 28 and 49, *Fop* was no longer detected (Fig. 7B and D). In addition, pea wilt disease did not occur in either field in the following crop season (2020). These results suggest that our specific detection technique will also be useful for evaluating the effectiveness of soil disinfestation.

It is currently necessary to take prompt and appropriate action against the outbreak of pathogens. The detection technique established in the present study may be used to minimize the damage caused by *Fop* by continuously monitoring fields with a history of disease outbreaks. The present study is not only important for the epidemiology of newly emerging pathogens, but also provides important insights into management of the *Fop* pathogen.

## Citation

Kotera, S., Hishiike, M., Saito, H., Komatsu, K., and Arie, T. (2022) Differentiation of the Pea Wilt Pathogen *Fusarium oxysporum* f. sp. *pisi* from Other Isolates of *Fusarium* Species by PCR. *Microbes Environ ***37**: ME21061.

https://doi.org/10.1264/jsme2.ME21061

## Supplementary Material

Supplementary Material

## Figures and Tables

**Table 1.** *Fusarium* isolates used in the present study

Species Form Isolate	Year	Place	Origin Plant	Source^a^	Reference	Pathogenicity in pea cv. Misasa^b^	Mating type^c^	GenBank Accession No.^d^	
								*TEF1α*	rDNA-IGS
*Fusarium oxysporum*									
f. sp. *pisi*									
1-1-M	2002	Aichi, Japan	Pea	YPPS	Sakoda *et al.* (2018)	+	1-2	LC648692*	LC648663*
1-2-1-5	2002	Aichi, Japan	Pea	YPPS		+	1-2	LC648693*	LC648664*
1-5-2-M	2002	Aichi, Japan	Pea	YPPS	Sakoda *et al.* (2019)	+	1-2	LC648694*	LC648665*
2-4-2-M	2002	Aichi, Japan	Pea	YPPS		+	1-2	LC648695*	LC648666*
2-9-M	2002	Aichi, Japan	Pea	YPPS		+	1-2	LC648696*	LC648667*
9-1-M-2	2003	Shizuoka, Japan	Pea	YPPS		+	1-1	LC648697*	LC648668*
10-1	2003	Shizuoka, Japan	Pea	YPPS		+	1-2	LC648698*	LC648669*
12-1	2003	Shizuoka, Japan	Pea	YPPS		+	1-2	LC648699*	LC648670*
KKB31	2015	Hokkaido, Japan	Pea	YPPS	Sakoda *et al.* (2019)	+	1-2	LC648691*	LC648662*
215B	2016	Wakayama, Japan	Pea	YPPS	Sakoda *et al.* (2019)	+	1-2	LC648685*	LC648656*
219A	2016	Wakayama, Japan	Pea	YPPS		+	1-2	LC648686*	LC648657*
22a	2016	Wakayama, Japan	Pea	YPPS		+	1-2	LC648687*	LC648658*
28a	2016	Wakayama, Japan	Pea	YPPS		+	1-2	LC648688*	LC648659*
39b	2017	Wakayama, Japan	Pea	YPPS	Sakoda *et al.* (2019)	+	1-2	LC648689*	LC648660*
49b	2017	Wakayama, Japan	Pea	YPPS		+	1-2	LC648690*	LC648661*
200929a	2020	Wakayama, Japan	Pea	TUAT	This study	+	1-2	LC648700*	LC648671*
f. sp. *adzukicola*									
241054	Unknown	Hokkaido, Japan	Adzuki bean	MAFF	Kondo *et al.* (2009)	–	1-1	LC648701*	LC648672*
f. sp. *apii*									
1017	Unknown	Japan	Celery	SUF		NT	1-2	LC648702*	AB106048
f. sp. *conglutinans*									
Cong:1-1	Unknown	Japan	Cabbage	TUAT	Kashiwa *et al.* (2013)	NT	1-1	LC648703*	AB106051
f. sp. *coriandrii*									
1709C2	2017	Ibaraki, Japan	Coriander	TUAT		NT	1-1	LC648704*	LC648673*
f. sp. *cubense* race 1									
160527	2016	Okinawa, Japan	Banana	TUAT	Nitani *et al.* (2018)	NT	1-2	LC648705*	LC648674*
f. sp. *cubense* tropical race 4									
FOC-BR		Indonesia	Banana	TUAT		NT	1-1	LC648706*	LC648675*
f. sp. *lycopersici* race 1									
103036	Unknown	Japan	Tomato	MAFF	Inami *et al.* (2014)	NT	1-1	LC648707*	AB106020
f. sp. *lycopersici* race 2									
103038	Unknown	Japan	Tomato	MAFF	Inami *et al.* (2014)	NT	1-1	LC648708*	AB106031
12575	Unknown	Tochigi, Japan	Tomato	JCM	Inami *et al.* (2014)	NT	1-1	LC648709*	AB106027
4287	Unknown	Spain	Tomato	Di Pietro	Di Pietro *et al.* (1998)	NT	1-1	KP693888	AB120973
f. sp. *lycopersici* race 3									
Chz1-A	Unknown	Kumamoto, Japan	Tomato	TUAT	Inami *et al.* (2014)	NT	1-2	LC648710*	AB373819
KoChi-1	Unknown	Kochi, Japan	Tomato	TUAT	Inami *et al.* (2012)	NT	1-1	LC648711*	AB675382
f. sp. *spinaciae*									
170612b	2017	Ibaraki, Japan	Spinach	TUAT		–	1-2	LC648712*	LC648676*
Other plant pathogenic isolates									
860926a	1986	Ibaraki, Japan	Mitsuba	TUAT		NT	1-1	LC648713*	LC648677*^e^
1709M	2017	Ibaraki, Japan	Mitsuba	TUAT		NT	1-1	LC648714*	LC648678*^e^
Non-pathogenic isolates									
K3-1	2017	Unknown	Pea	YPPS		–	1-1	LC648715*	LC648679*
K3-3	2017	Unknown	Pea	YPPS	Sakoda *et al.* (2018)	–	1-1	LC648716*	LC648680*
K4-1	2017	Unknown	Pea	YPPS		–	1-1	LC648717*	LC648681*
K4-2	2017	Unknown	Pea	YPPS		–	1-1	LC648718*	LC648682*
K5-2	2017	Unknown	Pea	YPPS	Sakoda *et al.* (2018)	–	1-1	LC648719*	LC648683*
Fo304	Unknown	Japan	Tomato	TUAT	Inami *et al.* (2014)	NT	1-1	LC648720*	AB373828*
*F. commune*									
f. sp. *rapae*									
ne-1	2017	Ibaraki, Japan	Potherb Mustard	TUAT		NT	1-1	LC648721*	LC648684*
Non-pathogenic isolate									
W5	2011	Aomori, Japan	Rice	TUAT	Saito *et al.* (2021)	–	1-1	LC648722*	LC516582*
*F. fujikuroi*									
Miyagi 92-10	Unknown	Miyagi, Japan	Rice	TUAT	Saito *et al.* (2021)	NT	1-1	LC648723*	LC649895*
*F. sacchari*									
7610	Unknown	Unknown		FGSC	Kawabe *et al.* (2005)	NT	1-2	LC648724*	AB106061
*F. solani*									
f. sp. *pisi*									
C1-2A	Unknown	Wakayama, Japan	Pea	TUAT		+	NA	LC648725*	NA
Other plant pathogenic isolate									
305125	Unknown	Unknown	Sweet pea	MAFF	Tsumuki *et al.* (1995)	–	NA	LC648726*	NA

^a^ YPPS, Yokohama Plant Protection Station; TUAT, Laboratory of Plant Pathology, Tokyo University of Agriculture and Technology; MAFF, Ministry of Agriculture, Forestry and Fisheries of the Japanese government; SUF, Shinshu University Fusarium collection; JCM, Japan Collection of Microorganisms; Di Pietro, Cordoba University; FGSC, Fungal Genetic Stock Center, Kansas State University. ^b^ +, pathogenicity; –, no pathogenicity; NT, not tested. ^c^ NA, no amplicon was obtained with EF1/EF2 primers for *TEF1α* and FIGS11/FIGS12 primers for rDNA-IGS. ^d^ Asterisks represent data obtained in this study. NA, no amplicon. ^e^ Not used for the phylogenetic ana­lysis due to a deletion of *ca.* 300 bp.

**Table 2.** Presence and absence of *SIX*s and *PDA1* genes in *Fusarium* isolates

Isolate^a^	PCR detection^b^														
	*SIX*														*PDA1*
	*SIX1*	*SIX2*	*SIX3*	*SIX4*	*SIX5*	*SIX6*	*SIX7*	*SIX8*	*SIX9*	*SIX10*	*SIX11*	*SIX12*	*SIX13*	*SIX14*	
*F. oxysporum*															
f. sp. *pisi*															
1-1-M (P1)	–^c^	–	–	–	–	LC648752^c^	–	–	–	–	–	–	LC648766	–	LC648734
1-2-1-5 (P3)	–	–	–	–	–	LC648753	–	–	–	–	–	–	LC648767	LC648781	LC648735
1-5-2-M (P1)	–	–	–	–	–	LC648754	–	–	–	–	–	–	LC648768	–	LC648736
2-4-2-M (P3)	–	–	–	–	–	LC648755	–	–	–	–	–	–	LC648769	LC648782	LC648737
2-9-M (P1)	–	–	–	–	–	LC648756	–	–	–	–	–	–	LC648770	–	LC648738
9-1-M-2 (P2)	–	–	–	–	–	–	LC648775	LC648776	–	LC648777	LC648778	LC648779	LC648771	LC648780	LC648739
10-1 (P1)	–	–	–	–	–	LC648757	–	–	–	–	–	–	LC648772	–	LC648740
12-1 (P1)	–	–	–	–	–	LC648758	–	–	–	–	–	–	LC648773	–	LC648741
KKB31 (P2)	–	–	–	–	–	LC648751	–	–	–	–	–	–	–	–	LC648733
215B (P1)	–	–	–	–	–	LC648745	–	–	–	–	–	–	LC648760	–	LC648727
219A (P1)	–	–	–	–	–	LC648746	–	–	–	–	–	–	LC648761	–	LC648728
22a (P1)	–	–	–	–	–	LC648747	–	–	–	–	–	–	LC648762	–	LC648729
28a (P1)	–	–	–	–	–	LC648748	–	–	–	–	–	–	LC648763	–	LC648730
39b (P1)	–	–	–	–	–	LC648749	–	–	–	–	–	–	LC648764	–	LC648731
49b (P1)	–	–	–	–	–	LC648750	–	–	–	–	–	–	LC648765	–	LC648732
200929a (P1)	–	–	–	–	–	LC648759	–	–	–	–	–	–	LC648774	–	LC648742
f. sp. *adzukicola*															
241054	–	–	–	–	–	–	–	–	–	–	–	–	+^c^	–	–
f. sp. *apii*															
1017	–	–	–	–	–	–	–	+	–	–	–	–	–	–	–
f. sp. *conglutinans*															
Cong:1-1	–	–	–	+	–	–	–	+	–	–	–	–	–	–	–
f. sp. *coriandrii*															
1709C2	–	–	–	–	–	–	–	–	–	–	–	–	–	–	–
f. sp. *cubense* race 1															
160527	–	–	–	–	–	+	–	–	–	–	–	–	+	–	–
f. sp. *cubense* tropical race 4															
FOC-BR	+	–	–	–	–	+	–	–	–	–	–	–	+	–	–
f. sp. *lycopersici* race 1															
103036	+	+	+	+	+	+	+	+	+	+	+	+	+	+	–
f. sp. *lycopersici* race 2															
103038	+	+	+	–	+	+	+	+	+	+	+	+	+	+	–
12575	+	+	+	–	+	+	+	+	+	+	+	+	+	+	–
4287	+	+	+	–	+	+	+	+	+	+	+	+	+	+	–
f. sp. *lycopersici* race 3															
Chz1-A	+	+	+	–	+	+	+	+	+	+	+	+	+	+	–
KoChi-1	+	+	+	+^d^	+	+	+	+	+	+	+	+	+	+	–
f. sp. *spinaciae*															
170612b	–	–	–	–	–	–	–	+	–	–	–	–	–	–	LC648743
Other plant pathogenic isolates															
860926a	–	–	–	–	–	–	–	+	–	–	–	–	–	–	–
1709M	–	–	–	–	–	–	–	+	–	–	–	–	–	–	–
Non-pathogenic isolates															
K3-1	–	–	–	–	–	–	–	–	–	–	–	–	–	–	–
K3-3	–	–	–	–	–	–	–	–	–	–	–	–	–	–	–
K4-1	–	–	–	–	–	–	–	–	–	–	–	–	–	–	–
K4-2	–	–	–	–	–	–	–	–	–	–	–	–	–	–	–
Fo304	–	–	–	–	–	–	–	–	–	–	–	–	–	–	–
*F. commune*															
f. sp. *rapae*															
ne-1	–	–	–	–	–	–	–	+	–	–	–	–	–	+	–
Non-pathogenic isolate															
W5	–	–	–	–	–	–	–	–	–	–	–	–	–	–	–
*F. solani*															
f. sp. *pisi*															
C1-2A	–	–	–	–	–	–	–	–	–	–	–	–	–	–	LC648744
Other plant pathogenic isolate															
305125	–	–	–	–	–	–	–	–	–	–	–	–	–	–	–

^a^ P1, P2, or P3 after the isolate number indicates a clade shown in Fig. 1. ^b^ Primers used are listed in Table S1. +, positive; –, negative. ^c^ +, amplicon obtained by PCR; –, no amplicon. Accession numbers indicate that the amplicon was present and sequenced, and data were deposited in GenBank. ^d^ This amplicon contains a transposon insertion (Inami *et al.*, 2012).

**Table 3.** Specific primer sets for the identification of *Fusarium oxysporum* f. sp. *pisi* isolates

Primer set	Primer name	Sequence (5′-3′)	Expected amplicon
piPDA	piPDAF	GGTCATTCTGAAAGAAGAGCTTCAGC	841 bp
	piPDAR	CCGTTGACACCAACCTCAGTCTGTTATC	
piSIX6	piSIX6F	GCTCCGTCTGCTATAAAGCCAATA	349 bp
	piSIX6R	GTCGATCCACCAATACCTTCATTC	
piSIX13	piSIX13F	ATCAGGCCTTCAACGAAGAG	739 bp
	piSIX13R	ATGGCGTTATGCTCATTGACACT	

**Table 4.** Detection with primer sets shown in Table 3

Isolate	Detection by PCR		
	piPDA	piSIX6	piSIX13
*Fusarium oxysporum*			
f. sp. *pisi*			
1-1-M	+	+	+
1-2-1-5	+	+	+
1-5-2-M	+	+	+
2-4-2-M	+	+	+
2-9-M	+	+	+
9-1-M-2	+	–	+
10-1	+	+	+
12-1	+	+	+
KKB31	+	+	–
215B	+	+	+
219A	+	+	+
22a	+	+	+
28a	+	+	+
39b	+	+	+
49b	+	+	+
200929a	+	+	+
f. sp. *adzukicola*			
241054	–	–	+
f. sp. *apii*			
1017	–	–	–
f. sp. *conglutinans*			
Cong:1-1	–	–	–
f. sp. *coriandrii*			
1709C2	–	–	–
f. sp. *cubense* race 1			
160527	–	–	+
f. sp. *cubense* tropical race 4			
FOC-BR	–	–	+
f. sp. *lycopersici* race 1			
103036	–	+	+
f. sp. *lycopersici* race 2			
103038	–	+	+
12575	–	+	+
4287	–	+	+
f. sp. *lycopersici* race 3			
Chz1-A	–	+	+
KoChi-1	–	+	+
f. sp. *spinaciae*			
170612b	+	–	–
Other plant pathogenic isolates			
860926a	–	–	–
1709m	–	–	–
Non-pathogenic isolates			
K3-1	–	–	–
K3-3	–	–	–
K4-1	–	–	–
K4-2	–	–	–
K5-2	–	–	–
Fo304	–	–	–
*F. commune*			
f. sp. *rapae*		
ne-1	–	–	–
Non-pathogenic isolate			
W5	–	–	–
*F. solani*			
f. sp. *pisi*		
C1-2A	–	–	–
Other plant pathogenic isolate			
305125	–	–	–

+, positive; –, negative.
