# Supplementary material for: Differentiation of the Pea Wilt Pathogen Fusarium oxysporum f. sp. pisi from Other Isolates of Fusarium Species by PCR
Source: Microbes Environ. 2022 Jan 1;37(1):ME21061. doi: 10.1264/jsme2.ME21061 (PMC8958301; doi:10.1264/jsme2.ME21061)
Supplement: Supplementary file 1 — Supplementary Material [file 37_21061_s1.pdf]

Table S1. List of primer sets used in this study

Target gene	Primer name	Sequence (5'-3')	Expected amplicon	Reference
TEF1α	EF-1<br>EF-2	ATGGGTAAGGAGGACAAGAC<br>GGAAGTACCAGTGATCATGTT	ca. 700 bp	O'Donell *et al.* (1998)
rDNA-IGS	FIGS11<br>FIGS12	GTAAGCCGTCCTTCGCCTCG<br>GCAAAATTCAATAGTATGGC	ca. 600 bp	Kawabe *et al.* (2005)
MAT1-1	Gfmat1a<br>Gfmat1b	GTTCATCAAAGGGCAAGCG<br>TAAGCGCCCTCTTAACGCCTTC	ca. 280 bp	Inami *et al.* (2012)
MAT1-2	GfHMG1<br>GfHMG2	TACCGTAAGGAGCGTCAC<br>GTACTGTCGGCGATGTTC	ca. 220 bp	Inami *et al.* (2012)
SIX1	P12-F2B<br>P12-R1	GTATCCCTCCGGATTTTGAGC<br>AATAGAGCCTGCAAAGCATG	992 bp	van der Does *et al.* (2008)
SIX2	SIX2-F2<br>SIX2-R2	CAACGCCGTTTGAATAAGCA<br>TCTATCCGCTTTCTTCTCTC	749 bp	van der Does *et al.* (2008)
SIX3	SIX3-F1<br>SIX3-R2	CCAGCCAGAAGGCCAGTTT<br>GGCAATTAACCACTCTGCC	608 bp	van der Does *et al.* (2008)
SIX4	SIX4-F1<br>SIX4-R1	TCAGGCTTCACTTAGCATAC<br>GCCGACCGAAAAACCCTAA	967 bp	van der Does *et al.* (2008)
SIX5	SIX5-F1<br>SIX5-R1	ACACGCTCTACTACTCTTCA<br>GAAAACCTCAACGCGGCAAA	667 bp	van der Does *et al.* (2008)
SIX6	SIX6-F<br>SIX6-R	GGCTGCGTAGCTGGTCCCCT<br>CATGTCATGAATGTACGCATGTCCCT	611 bp	Meldrum *et al.* (2012)
SIX7	SIX7-F1<br>SIX7-R1	CATCTTTTCGCCGACTTGGT<br>CTTAGCACCCTTGAGTAACT	862 bp	Lievence *et al.* (2009)
SIX8	SIX8-F<br>SIX8-R	TCGCCTGCATAACAGGTGCCG<br>TTGTGTAGAAACTGGACAGTCGATGC	250 bp	Meldrum *et al.* (2012)
SIX9	FOL SIX9 F<br>FOL SIX9 R	GGGTGGACCATATCACGATGTTCG<br>GAATACCTGAGTGGAGTTGTGTCTTG	458 bp	Taylor *et al.* (2016)
SIX10	SIX10 F<br>SIX10 R	GTTAGCAACTGCGAGACACTAGAA<br>AGCAACTTCCTTCCTCTTACTAGC	636 bp	Taylor *et al.* (2016)
SIX11	SIX11 F<br>SIX11 R	ATTCCGGCTTCGGGTCTCGTTTAC<br>GAGAGCCTTTTTGGTTGATTGTAT	559 bp	Taylor *et al.* (2016)
SIX12	SIX12 F<br>SIX12 R	CTAACGAAGTGAAAAGAAGTCCTC<br>GCCTCGCTGGCAAGTATTTGTT	449 bp	Taylor *et al.* (2016)
SIX13	SIX13 F<br>SIX13 R	CCTTCATCATCGACAGTACAACG<br>ATCAAACCCGTAACTCAGCTCC	1027 bp	Taylor *et al.* (2016)
SIX14	SIX14 FOL F<br>SIX14 FOL R	ATAAAGTGCGACTGGACTTCTGCC<br>ACCCCCATCCACATTCCTAAGCGA	421 bp	Taylor *et al.* (2016)
PDA1	PDAF2a<br>PDAR3a	ACCCATTGTTYGYATAGGRCCG<br>ATCCGTTGACACCAACCTCAGT	1086 bp	Milani *et al.* (2012)

Table S2. List of non-Japanese *Fusarium oxysporum* f. sp. *pisi* isolates used in the phylogenetic analysis

Species<br>Form<br>Isolate	Place	Plant	Source[a]	GenBank Accession No.<br>*TEF1α*	Reference
Fusarium oxysporum					
f. sp. *pisi*					
170.3	USA	Pea	CBS	MT630357	Jenkins *et al.* (2021)
F16	USA	Pea		MT630365	Jenkins *et al.* (2021)
F231	USA	Pea		MT630361	Jenkins *et al.* (2021)
F232	USA	Pea		MT630366	Jenkins *et al.* (2021)
F233	USA	Pea		MT630371	Jenkins *et al.* (2021)
F234	USA	Pea		MT630363	Jenkins *et al.* (2021)
F235	USA	Pea		MT630364	Jenkins *et al.* (2021)
F236	USA	Pea		MT630367	Jenkins *et al.* (2021)
F237	USA	Pea		MT630370	Jenkins *et al.* (2021)
F30	USA	Pea		MT630369	Jenkins *et al.* (2021)
F31	USA	Pea		MT630362	Jenkins *et al.* (2021)
F35	USA	Pea		MT630368	Jenkins *et al.* (2021)
F40	USA	Pea		MT630373	Jenkins *et al.* (2021)
F42a	USA	Pea		MT630375	Jenkins *et al.* (2021)
F79	USA	Pea		MT630356	Jenkins *et al.* (2021)
F81	USA	Pea		MT630360	Jenkins *et al.* (2021)
FOP1 EMR	UK	Pea		MT630382	Jenkins *et al.* (2021)
FOP2	UK	Pea		MT630383	Jenkins *et al.* (2021)
Fw-09-C	USA	Pea		MT630358	Jenkins *et al.* (2021)
Fw-09-D	USA	Pea		MT630359	Jenkins *et al.* (2021)
PDA3b	USA	Pea		MT630374	Jenkins *et al.* (2021)
R2	Czech Republic	Pea		MT630372	Jenkins *et al.* (2021)
FOP5	UK	Pea		KP964891	Taylor *et al.* (2016)
36311	UK	Pea	NRRL	KP964898	Taylor *et al.* (2016)

[a] CBS, Dutch Centraalbureau voor Schimmelcultures, Fungal Biodiversity Centre, Netherlands; NRRL, Northern Regional Research Lab, Agriculture Research Service Culture Collection of the United States Department of Agriculture, Peolia, IL USA

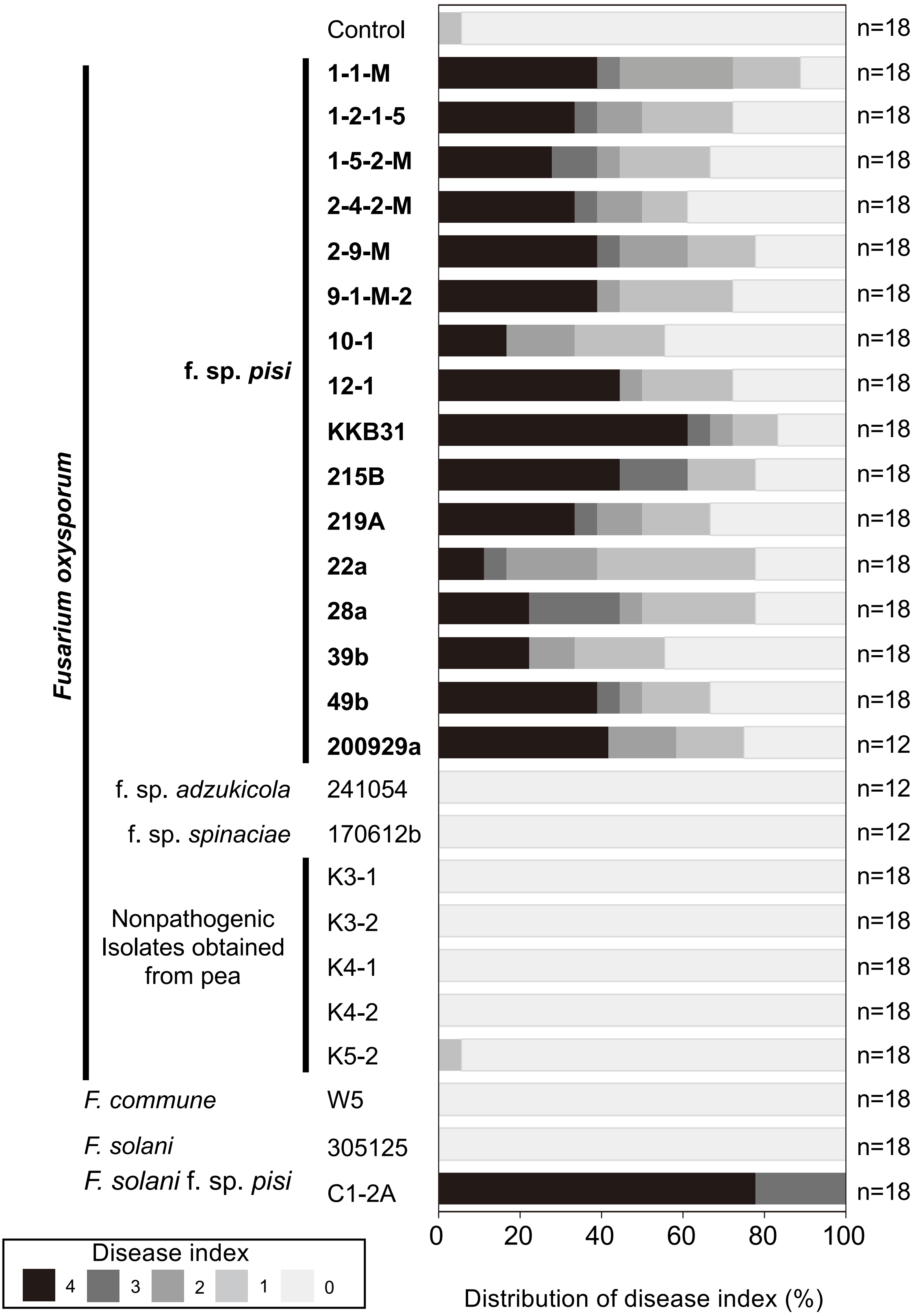

Fig. S1. Pathogenicity of the *Fusarium oxysporum* f. sp. *pisi* isolates

Pea (cv. Misasa) plants were inoculated with the 16 Japanese *Fop* isolates and 10 isolates of other Fusaria by drenching soil with 1 ml of the bud-cell suspension (1.0×10^7 cells/ml) of each isolate. Sterilized water was used as a control. Each plant was evaluated 28 days post inoculation. The stacked graphs represent totals of 12 or 18 plants per inoculum, showing the severity of disease in each individual rated by external symptoms on a 0 to 4 scale. External symptoms were rated as follows: 0, no symptoms; 1, yellowing or wilting of the lower leaves; 2, yellowing or wilting of the lower and upper leaves; 3, wilting of the entire plant; 4, death.
